# Deep Palmar Hand Infections

**Published:** 1918-12

**Authors:** 


**Deep Palmar Hand Infections.** By HOWARD L. BEYE, M.D. *Annals of Surgery.* February 1918.

The author simply discusses the infections lying deeper than the palmar fascia. The most important diagnostic features are: first, the local areas of tenderness; second, the pain elicited by passive movements of the fingers and hand; third, the presence of a wound which is generally the initial source of infection.

*Tenderness.* The determination of the areas of tenderness necessitates a very long, tedious and painful examination. A blunt instrument proves most convenient for palpation. The fact that all the hand is always more or less tender, and that the reaction to the painful sensations will be essentially different according to each patient, should constantly be kept in mind by the surgeon.

*Pain on Motion.* Each finger and the thumb must be examined separately, and carefully, and discrimination must be made between the movements that prove more or less painful. Extension and flexion of the hand at the wrist must also be examined.

*Local Signs of Infection.* A local wound will often account for infection, and give a clue to the localization of the deeper process.

Edema and redness of the skin are usually limited to the back of the hand, and this localization has been responsible for numberless incisions on the dorsum of the hand in cases in which not a drop of pus was found except in the palm.

*General Symptoms.* These are the general symptoms of any pyogenic infection; however, cases have been dealt with in which they failed completely, especially in low grade infections.

*Localization of infection.*

a) *Ulnar Bursa.* Hand held semiflexed at wrist and with fingers and thumb partially flexed. Tenderness is definitely localized over the ulnar bursa, from the tip of the little finger to an inch above the wrist-joint. Pain is elicited by extension of the little finger. Redness is often present on the palmar aspect of the wrist, just proximal to the annular ligament, and when present is an extremely important sign of infection of the ulnar bursa. It extends around the wrist, as a band, about a finger's breadth in width; is somewhat edematous, and very tender b) *Radial Bursa.* Tenderness localized over the tendon of the m. flexor pollicis longus, from the tip of the thumb to about an inch above the wrist-joint. Severe pain produced by extension of the thumb, referred along the entire tendon into the forearm. Severe pain elicited by extension of the hand at the wrist. Edema and redness of the palmar aspect may be present.

c) *Middle Palmar Space.* Tenderness is localized in the middle of the palm, extends only up to the annular ligament, and is not present in the wrist. Pain is increased by extension of the middle and ring fingers. Extension of the wrist will cause only slight pain if the fingers are flexed. Swelling and redness on the dorsum of the hand are usually very marked.

d) *Palmar Thenar Space.* Tenderness is localized in the palm over the space. It does not extend above the annular ligament nor on the thumb. Pain is elicited by extension of the index finger. Bulging in the radial half of the palm is quite characteristic of infection in this space. Edema and redness on the back of the hand is present and most marked over the radial half e) *Dorsal Thenar Space.* Tenderness most marked on the dorsal aspect of the hand, between the first and second metacarpal bones. Pain is best elicited by abduction of the thumb.

*Treatment.* Deep hand infections should be considered as emergency surgical cases. A delay of only a few hours may make a great difference in the infection. The author considers it wiser, if there is doubt as to whether or not infection is present in a certain space, to explore that space, rather than to wait and see what may happen.

A general anesthetic is advisable, ether being the anesthetic of choice. A constrictor should be used as it allows of careful scrutiny of the bloodless tissues. Its use is contraindicated if there is a lymphangitis extending as high as the elbow. Tincture of iodine should be used to render the whole hand and forearm aseptic.

The plates accompanying the paper show exactly where each space should be incised for the pus.

*Ulnar Bursa* must be widely opened from the distal phalanx of the fifth finger to the distal border of the annular ligament of the wrist. The best drainage is obtained by complete incising of that ligament, but prolapse of the tendons is to be feared after that incision. In many cases sufficient drainage will be obtained by leaving the ligament wholly or partially intact.

The greatest amount of pus is often found in that part of the ulnar bursa lying *behind* the tendons in the wrist and forearm. To drain this, an incision may be practised on the ulnar side of the forearm hugging the anterior surface of the ulna.

*Radial Bursa.* One incision should extend from the distal phalanx of the thumb to within a finger's breadth of the annular ligament. Another incision about an inch wide is practised on the anterior surface of the wrist proximal to the annular ligament. A third one, about 2 inches long, should extend along the anterior border of the radius.

The branch of the median nerve passing to the thenar group of muscles must be carefully spared, even at the expense of insufficient drainage.

*Middle Palmar Space.* The web between middle and ring fingers is split, and the incision retracted by spreading the fingers.

*Palmar Thenar Space.* The incision should begin just above the web between the thumb and index finger, and be carried up for about an inch parallel to the first metacarpal bone.

In all cases the drainage is maintained by a gutta percha strip laid as deeply as possible in the incision, and carried to the highest point of the infected space. After the drains are placed the constrictor is removed. Ligature is rarely needed and the bleeding soon stops. If the annular ligament has been completely incised, the arm must be dressed on a dorsal board splint with the wrist and fingers held flat against it.

Hot boric dressings are applied during the first hours. They are replaced by plain gauze dressings spread thinly with sterile vaseline. Drains are removed after 48 hours.

After the third day, the hand is soaked every day for 15 minutes in a hot bath to which is added tincture of iodine. Passive motion of the fingers is gently begun during that bath, and the movements gradually extended as far as possible.

*Results.* When the radial and ulnar bursae are drained for infection throughout their extent, there is never a 100 per cent. recovery of function. Contraction of the little finger is frequent even under the best conditions of treatment.

Return of function after uncomplicated infection of the middle palmar space, palmar thenar, and dorsal thenar space, may approximate 100 per cent.

